# Current and Future Trends in Otorhinolaryngology–Head and Neck Surgery

**DOI:** 10.3390/jcm14227935

**Published:** 2025-11-09

**Authors:** Konstantinos Chaidas

**Affiliations:** 1 Ear, Nose, and Throat Department, University Hospital of Alexandroupolis, School of Medicine, Democritus University of Thrace, 68100 Alexandroupolis, Greece; konchaidas@gmail.com; 2 Ear, Nose, and Throat Department, Imperial College Healthcare NHS Trust, London W2 1NY, UK

Over the past decade, there has been a significant increase in available knowledge in the specialty of otorhinolaryngology–head and neck surgery with a remarkable development in diagnostic and therapeutic modalities. As a result of continuous advances in digital technologies, their integration into clinical practice has become a notable trend. Telemedicine proved to be an effective tool in ENT clinics, especially during the COVID-19 pandemic due to associated restrictions in face-to-face consultations [1]. Furthermore, machine learning algorithms are increasingly being used for data and image analysis with the aim to improve diagnostic accuracy and outcome prediction [2,3,4]. At the same time, the adoption of minimally invasive and function-preserving techniques continues to improve patient safety and recovery times while maintaining excellent results. Namely, thermal ablation is currently used not only for the management of benign thyroid nodules, but also for patients with papillary thyroid microcarcinoma with satisfactory outcomes [5]. Likewise, the role of transoral robotic surgery in selected patients with obstructive sleep apnea or head and neck cancer has been established. The use of a surgical robot provides better surgical access and dexterity, satisfactory outcomes, and reduced postoperative morbidity [6,7]. Of course, a multidisciplinary patient-centered approach is vital to ensuring the provision of high-quality care for all patients.

Our Special Issue, “Current and Future Trends in Otorhinolaryngology–Head and Neck Surgery”, aims to provide readers with the latest developments in the field of otorhinolaryngology–head and neck surgery and underscore current and future trends. The included contributions cover a wide range of topics reflecting both clinical innovation and translational research.

The COVID-19 pandemic has significantly affected medical practice in general and ENT specialty in particular, as demonstrated in this Special Issue (Contributions 1–3). Some of the studies evaluated the use of new diagnostic methods for patients with nasal dysfunction (Contributions 4, 5). Moreover, innovative treatment strategies were explored, with a number of articles assessing the efficacy of new techniques or the current role of traditionally used techniques (Contributions 6–9). In addition, the valuable experience of certain institutions in the management of challenging clinical problems was shared (Contributions 10–14), with the role of diagnostic biomarkers also investigated (Contributions 15, 16). Finally, the evolving trends and future demands in ENT procedures were reported (Contribution 17).

In summary, this Special Issue highlights the innovation of the global community of otorhinolaryngology–head and neck surgery, illustrating how technological advancements, increasing scientific knowledge, and clinical excellence converge to shape the future of our specialty. Ultimately, continuous collaboration between researchers and clinicians is essential for improving patient care.
